# Relationship Between the Changes in the Inclination of the Incisors and Soft Gingival Tissue Remodeling During the First Phase of Orthodontic Treatment Without Premolar Extraction

**DOI:** 10.3390/dj13120587

**Published:** 2025-12-08

**Authors:** Oleksandr Kobylyanskyy, Marco Aoqi Rausch, Alina Kobylyanska, Oleh Andrukhov, Xiaohui Rausch-Fan

**Affiliations:** 1 Center for Clinical Research, University Clinic of Dentistry, Medical University of Vienna, 1090 Vienna, Austria; ortho.composer@gmail.com (O.K.); xiaohui.rausch-fan@meduniwien.ac.at (X.R.-F.); 2 International School of Progressive Orthodontics, 01135 Kyiv, Ukraine; lyutyanskaya.lina@gmail.com; 3 P.L. Shupyk National Academy of Postgraduate Education Ukraine, 01001 Kyiv, Ukraine; 4 Clinical Division of Orthodontics, University Clinic of Dentistry, Medical University of Vienna, 1090 Vienna, Austria; marco.rausch@meduniwien.ac.at; 5 Competence Center for Periodontal Research, University Clinic of Dentistry, Medical University of Vienna, 1090 Vienna, Austria; 6 Clinical Division of Periodontology, University Clinic of Dentistry, Medical University of Vienna, 1090 Vienna, Austria

**Keywords:** orthodontic treatment, alveolar bone, gingival recession, clinical crown height, gingival thickness, dehiscence, proclination, retro-inclination

## Abstract

**Background/Objectives**: The present study aimed to investigate how the changes in the inclination of the upper and lower incisor regions during the alignment phase of orthodontic treatment (OT) without premolar extraction influence the height and thickness of the gingiva around these incisors. **Methods**: This prospective clinical study included 62 patients undergoing OT without premolar extraction. Cone beam computed tomography and intraoral 3D scans were taken before and after the alignment phase, and superimposed using specialized software. The changes in the inclination of each tooth, alveolar bone height (ABH), clinical crown height (CCH), and gingival thickness on the level of the cement–enamel junction (CEJ) and 1 mm below it were determined on both tooth sides. **Results**: The alignment phase of OT was accompanied by an increase in CCH on the buccal side of both upper and lower incisors. In contrast, on the palatal/lingual sides, either a decrease or an increase in CCH was observed, depending on the direction and amount of the changes in the inclination. Furthermore, in many cases, a decrease in the gingival thickness was observed, which was especially pronounced on the palatal side of the upper incisors after proclination. The changes in the CCH and gingival thickness on the palatal/lingual sides exhibited a weak to moderate correlation with the changes in the inclination. In addition, a very weak to weak correlation between the changes in gingival parameters and ABH was observed. **Conclusions**: Our data suggest that there is some soft tissue remodeling during the alignment phase of OT, and the changes in soft tissue parameters exhibit some dependency on the changes in inclination. Potential changes in the soft tissue should be considered in planning orthodontic treatment.

## 1. Introduction

Orthodontic treatment (OT) is aimed at correcting tooth malocclusion, which requires both bone and soft gingival tissue remodeling and needs to be thoroughly planned [1,2,3]. The significant risks during OT are gingival recession and dehiscence [4]. Gingival recession (GR) refers to the migration of the gingival margin apically of the cementoenamel junction [5]. Exposing the root surface in the area of the incisors can lead to tooth hypersensitivity, esthetic deterioration, and root caries [6,7,8]. GR appears only in areas of alveolar bone defects [9], but its etiology is unknown and thought to have a multifactorial origin. In the initial stages, GR can be indicated by an increase in the clinical crown height (CCH), defined as the distance between the incisal edge and the free gingival margin [10]. The risk of GR appearance after orthodontic treatment is increased, especially on the buccal side, after the vestibular inclination of incisors with a high amplitude [11,12]. Dehiscence is defined as an apical alveolar bone displacement of more than 2 mm relative to the cementoenamel junction (CEJ) [13]. Alteration of the alveolar bone height during OT and the appearance of dehiscence depend on the direction and extent of changes in tooth inclination [14].

The interconnection between the apical migration of the gingival margin and the alveolar bone remodeling during OT is not straightforward: GR does not develop in all teeth with existing dehiscence, and an important risk factor here is gingival thickness [1]. Thin gingiva is characterized by a small amount of keratinized gingiva, a scalloped gingival contour, and a thin bone base, and it is more susceptible to inflammation and trauma than the thick biotype [15,16]. Patients with thin gingiva are more susceptible to apical gingiva displacement, even without OT [17,18,19]. Excessive inclination of lower incisors with a thin gingival biotype during OT results in more frequent and pronounced recessions [1]. Interestingly, changes in inclination with a high amplitude can also result in a higher prevalence of a thin gingival biotype buccally after OT [20].

Optimal OT planning should also assess possible risk factors for dehiscence and GR and prevent their occurrence. To achieve this, it is essential to understand how bone and soft tissue are altered during orthodontic tooth movement in different directions and amplitudes, but data on this topic remain limited. Regarding soft tissue remodeling, it is essential to control CCH and gingival thickness throughout OT. Some earlier publications did not find any effect of changing the inclination of the incisors on the vertical level of alveolar bone and gingival margin migration, but most of these studies were retrospective, used classical clinical or instrumental 2D diagnostic methods, and focused on the state of the periodontium after completion of orthodontic treatment [21,22,23,24,25,26,27,28,29]. Furthermore, there is a lack of information on changes in soft tissues across different stages of OT, including the relationship between bone and soft tissue changes and how different amplitudes and directions of incisor displacement affect these processes.

The development of diagnostic 3D technologies enables a more detailed assessment of the change in periodontal parameters during OT. Using cone beam computed tomography (CBCT), it is possible to determine the direction and amount of inclination change for each incisor separately, unlike previous 2D research methods, which determined the direction and amount of displacement for all incisors at once [14]. This method can be quite inaccurate, especially in a crowded environment of teeth, and can over- or underestimate the changes in a single tooth’s inclination. The application of intraoral scanners for measuring the position of the marginal gingival edge relative to the incisal edge is also advantageous compared to previous methods, such as plaster models, calipers, or evaluating photographs [30,31,32]. Using the latest software, it is possible to superimpose hard and soft periodontal tissues [12,33]. This allows for the non-invasive measurement of gingival height and thickness not only buccally but also palatally/lingually, eliminating patient discomfort and the inaccuracies that may occur during the clinical assessment of gingival parameters. However, superimposition of CBCT and oral scanning have never been used to assess soft gingival tissue remodeling depending on the tooth inclination changes during OT.

This study aims to determine the effect of changes in individual incisor inclination on the thickness and position of the marginal gingiva buccally and palatally/lingually in the area of the upper and lower incisors during the incisor alignment phase of orthodontic treatment without premolar extraction. A further aim was to evaluate how these changes in the soft tissue correlate with the vertical changes in alveolar bone, which were analyzed in detail in our recent study [14].

## 2. Materials and Methods

### 2.1. Ethics Considerations

This study was approved by the Research Ethics Committee of P.L. Shupyk National Academy of Postgraduate Education, Ukraine (Protocol 11 from 3 November 2014). Informed consent was obtained from all patients and/or their parents for study participation.

### 2.2. Study Population

62 patients (43 females and 19 males) were included in this prospective clinical study, with a mean age of 25.9 ± 9.6 years. Patients had OT at the private clinic “Implantological Center” (Kyiv, Ukraine) between January 2017 and December 2021 and were treated for complaints of an unaesthetic smile caused by dental crowding to correct the bite. Inclusion criteria: a permanent dentition, the need for OT, being >14 years of age, and written consent to participate in the study. In addition, patients or their representatives signed informed consent for the OT plan. Exclusion criteria: the absence of teeth in the anterior region, severe crowding of the incisors (>4 mm), anterior crossbite, multiple missing teeth, history of dental trauma of incisors, use of removable dentures, presence of implants, crowns, veneers, or cervical restorations in the anterior region of the upper and lower jaws, attached gingiva height lower than 1 mm in the anterior region, prior surgical interventions on jaws and gums, periodontitis, gingival recession, cleft lip or palate, lip or tongue piercing, current smoking, consumption of narcotic drugs, presence of systemic diseases, impaired nasal breathing, allergic reactions to orthodontic materials, pregnancy or lactation and mental disorders or depression. All incisors were included in the analysis; thus, the study includes 248 upper incisors and 248 lower incisors.

### 2.3. Orthodontic Treatment

Patients had different indications for OT; most of them had a distal bite. A stepwise treatment protocol was followed to establish a proper alignment and a more efficient and effective treatment process. The straight-wire technique protocol was used for OT. It was initiated by employing highly elastic leveling archwires to level out the teeth’s horizontal, vertical, and rotational positional discrepancies [34]. For OT, the McLaughlin-Bennett-Trevisi appliance with a 0.022-inch slot was used, manufactured by 3M Unitek (Monrovia, CA, USA). Depending on the esthetic preferences of the patients, ceramic (Clarity advanced) or metal (Gemini) brackets were used, along with buccal tubes for all molars. The resin composites ‘Transbond XT’, manufactured by 3M Unitek, were used for bonding braces. All patients received training and advice on personal oral hygiene while wearing braces before the start of OT and were regularly monitored throughout the treatment process. No visible signs of inflammation were mentioned during the treatment.

During the initial treatment phase, aligning the teeth, the orthodontic wires were gradually replaced with ascending dimensions. Depending on the clinical situation and the rate of tooth movement, the wire replacement was performed at intervals of 1 to 4 months. The sequence of wires used was as follows: 0.014, 0.016, and then 0.018-inch nitinol round wires, followed by rectangular (0.016–0.022 inch) nitinol heat-activated archwires (all 3M Unitek). When the incisors in both jaws were aligned and a 0.016–0.022-inch stainless steel wire had been in use for at least 1 month, the alignment phase was considered complete. Occlusion and spaces between teeth were corrected during the second phase of the treatment and are beyond the scope of the present manuscript. OT were performed by the same dentist (O.K.). The mean duration of the aligning phase was about 9.3 months and varied between individual patients.

### 2.4. Determination of the Individual Incisor Inclinations

The individual inclination of individual incisors was determined as described in a recent study [14]. The specific reasons for which CBCTs were taken were as follows: intent to use orthodontic miniscrews, extraction of the third molars, etc. All patients and their parents signed informed consent. Three-dimensional CBCT scans were taken using a Veraviewepocs 3D R100 device (J. Morita Mfg. Corp., Kyoto, Japan) before the start of OT (CBCT1) and after the alignment phase (CBCT2), as shown in the supplementary information of our recent study [14]. CBCT scan settings were as follows: 80 kV, 5 mA, 9.4 s exposure time, 0.125 mm voxel resolution and 80–80 mm FOV. Reconstruction of the CBCT data was accomplished at 0.125 mm increments and further analyzed using the ‘i-Dixel 2.0’ software (J. Morita Mfg. Corp., Kyoto, Japan). The field of view (FOV) of the CBCT scans was restricted to the upper and lower incisors to minimize radiation exposure. All CBCTs were performed by an experienced radiologist, who trained the operator to analyze CBCTs to evaluate various bone parameters. For evaluation of the individual inclination of the incisors, CBCT1 and CBCT2 were superimposed as previously described [14]. Measurements were made in the sagittal plane; the section with the best determination of tooth axis quality was selected using axial-guided navigation [14,35]. The severity and direction of the displacement during initial OT were determined by the difference in tilt angles between CBCT1 and CBCT2. Positive and negative values indicated a buccal displacement and a palatal or lingual inclination, respectively. Teeth were assigned into three groups: “Retro-inclination” (lingual crown inclination < 0°), “Proclination-low” (buccal crown inclination between 0° and 5°), or “Proclination-high” (buccal crown inclination > 5°).

### 2.5. Measurement of the Bone Parameters

The assessment of three-dimensional (3D) bone architecture was performed as described in our recent study [14]. Measurements of the vertical position of the alveolar bone were conducted in the sagittal section along the center of the tooth on the buccal and palatal/lingual sides of the upper and lower incisors. The sagittal section, passing from the midpoint of the incisal edge to the root apex, was used for evaluation.

### 2.6. Measurement of the Gingival Parameters

A Trios color intraoral 3D scanner (3Shape, Copenhagen, Denmark) was used to scan the patient’s teeth and gingiva before and after the alignment phase (scan1 and scan2, respectively). Before use, the scanner was calibrated as directed by the manufacturer’s guidelines. All scans were performed by a single trained investigator. A consistent scanning environment was maintained by scanning with the dental light off and the windows closed. Approximately 1700 ± 100 images were captured during cast scanning (mean scanning time of 78 s). Manufacturer-recommended scanning distances and strategies were adhered. Scans 1 and 2 were exported as stereolithography (STL) files, STL1 and STL2, respectively. Image reconstruction for visual analysis was performed using the Exocad software (Exocad GmbH, Darmstadt, Germany). Intraoral STL1 and STL2 scans were then merged with CBCT1 and CBCT2 accordingly (Supplementary Figure S1). Automatic alignment of STL and Digital Imaging and Communications in Medicine (DICOM) files was performed.

Clinical crown height (CCH) was measured as the distance between the incisal edge and the point of the free gingival margin [10] and performed on each incisor of the upper and lower jaws, buccally and palatally/lingually (Supplementary Figure S2).

The gingival thickness (GTH) measurements were conducted on the buccal and palatal/lingual sides of the upper and lower incisors in the sagittal section along the center of the tooth. The sagittal section, passing from the midpoint of the incisal edge to the root apex, was used for evaluation. The gingival thickness at CEJ (GTH_CEJ) and 1 mm apically from the CEJ (GTH_1mm) was evaluated for each tooth (Supplementary Figure S2). All sites were measured by the same dental technician (computer-aided design (CAD) designer) and were performed in duplicates.

### 2.7. Statistical Analysis

Data are presented as mean ± SD. Normality was assessed using the Kolmogorov–Smirnov test. Since the normal distribution was not observed across all groups, nonparametric tests were used for all comparisons. Changes in soft tissue parameters during the alignment phase were evaluated using the Wilcoxon test. The Kruskal–Wallis test was utilized to assess differences between the groups “Retro-inclination”, “Pro-inclination low”, and “Pro-inclination high”, followed by the Mann–Whitney U test for pairwise comparisons. The correlation between metric parameters was tested by the Spearman method. All statistical analyses were performed using SPSS 27.0 software (IBM, Armonk, NY, USA). Differences were considered statistically significant if the *p*-value was less than 0.05. The graphs were created in GraphPad Prism version 10.6.1 (La Jolla, CA, USA).

Power analysis was performed using a freely available sample size calculator (https://homepage.univie.ac.at/robin.ristl/samplesize.php, assessed on 12 November 2025), assuming the level of α = 0.05. To characterize the effect size, Cohen’s d was calculated. For the comparisons using the Wilcoxon test between time points T1 and T0, Cohen’s d was calculated by dividing the mean values of changes in the target parameters by the standard deviation of these changes. For the comparison of the independent variables by the Mann–Whitney U test, Cohen’s d was calculated using a freely available calculator (https://www.socscistatistics.com/effectsize/default3.aspx, assessed on 12 November 2025). The effect size was estimated as small, medium, or large if Cohen’s d was 0.2, 0.5, or 0.8, respectively [36].

## 3. Results

**Figure 1:**
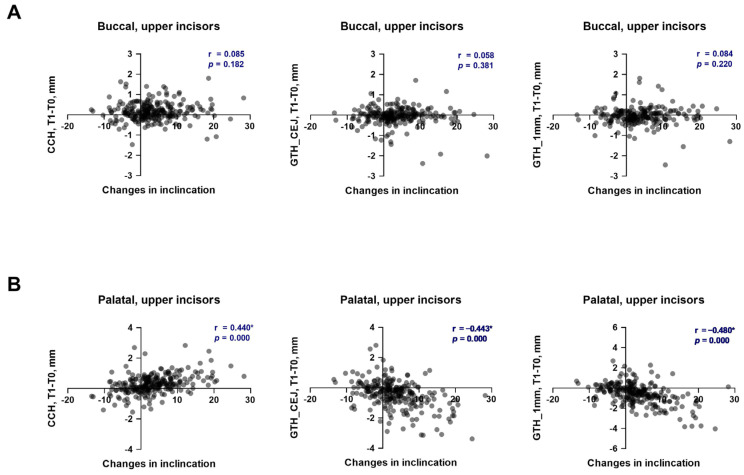
Relationship between the changes in soft tissue parameters and alteration of tooth inclination in upper incisors during the first phase of orthodontic treatment. (**A**)—buccal side; (**B**)—palatal side. Each point represents the values measured on an individual tooth. For each pair of parameters, a correlation coefficient (r) and *p* values are calculated using the Spearman method. Correlations with *p* < 0.05 were considered to be statistically significant. *—statistically significant correlation.

**Figure 2:**
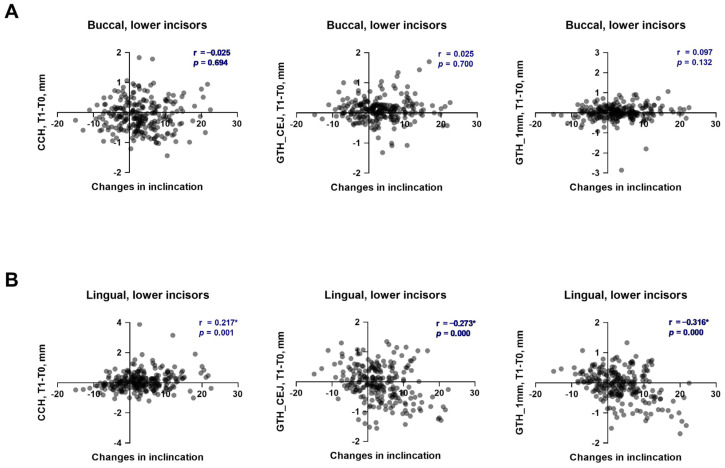
Relationship between the changes in soft tissue parameters and alteration of tooth inclination in lower incisors during the first phase of orthodontic treatment. (**A**)—buccal side; (**B**)—lingual side. Each point represents the values measured on an individual tooth. For each pair of parameters, a correlation coefficient (r) and *p* values were calculated using the Spearman method. Correlations with *p* < 0.05 were considered to be statistically significant. *—statistically significant correlation.

The soft tissue parameters before and after the alignment phase of OT, along with their changes depending on the direction and magnitude of individual inclination alterations in the upper and lower incisors, are summarized in Table 1 and Table 2, respectively. The relationships between the changes in soft tissue parameters and the alteration in the inclination of upper and lower incisors are presented in Figure 1 and Figure 2, respectively. Cohen’s d and statistical power for statistically significant comparison in the upper and lower incisors are provided in the Supplementary Tables S1 and S2, respectively.

### 3.1. Changes in Soft Tissue Parameters Depending on the Alteration in the Inclination of the Upper Incisors

After the first phase of OT, CCH increased on both the buccal and palatal sides of the upper incisors, regardless of the direction and magnitude of the tooth movement (Table 1). All changes were statistically significant on the buccal side, but no differences between groups with different directions and amounts of tooth movement were observed. No correlation between the changes in CCH on the buccal side and individual inclination of upper incisors was observed (Figure 1A, r = 0.058). On the palatal site, only proclination resulted in significant changes in CCH during the first OT phase. Furthermore, the changes in CCH observed in the “Proclination-high” group were significantly higher than those in the “Proclination-low” group. A moderate, significant positive correlation between the changes in CCH on the palatal side and individual inclination of upper incisors was observed (Figure 1B, r = 0.440). Most of the observed statistically significant differences in the CCH changes during the first OT phase and the differences in the CCH changes between various groups exhibited a statistical power higher than 0.8, and the effect size was small to medium for the buccal side and small to large for the palatal side (Table S1).

Gingival thickness reduced during OT on both buccal and palatal sides of upper incisors, regardless of the direction and magnitude of tooth movement. However, on the buccal side, these changes were less than 0.1 mm, and significant changes were observed only at the level of 1 mm from CEJ in the “Proclination-low” group. No correlation between the changes in the gingival thickness on both levels and the individual inclination of upper incisors was observed (Figure 1A, r = 0.084 and r = 0.085). Palatally, gingival thickness at both evaluated levels was significantly reduced in “Proclination-low” and “Proclination-high” groups. Moreover, the changes in gingiva thickness were significantly higher in the “Proclination-high” group compared to the “Proclination-low” group. At both evaluated levels, a moderate, significant negative correlation between the changes in gingiva thickness on the palatal side and individual inclination of upper incisors was observed (Figure 1B, r = −0.480 and r = −0.440). Most of the observed statistically significant differences in the CCH changes during the first OT phase and the differences in the gingiva thickness changes between various groups exhibited a statistical power higher than 0.8, and the effect size in most cases was medium to large for the palatal side (Table S1).

### 3.2. Changes in Soft Tissue Parameters Depending on the Alteration in the Inclination of the Lower Incisors

After the first phase of OT, CCH on the buccal side significantly reduced in the “Proclination-low” group and significantly increased in the “Proclination-high” group. However, no significant difference in the CCH changes during the first phase of OT was observed between groups with different alterations in inclination. Furthermore, no correlation was observed between changes in CCH on the buccal side and the inclination of individual lower incisors (Figure 2A, r = 0.025). On the lingual side of lower incisors, CCH was significantly reduced in the “Retro-inclination” group and significantly increased in both the “Proclination-low” and “Proclination-high” groups. Furthermore, the changes in CCH observed in the “Proclination-high” group were significantly higher than those in the “Proclination-low” group. A weak, significant positive correlation between the changes in CCH on the lingual side and individual inclination of lower incisors was observed (Figure 2B, r = 0.217). Most observed statistically significant differences in CCH changes in the lower incisors during the first OT phase and between groups did not achieve the 0.8 power level, and the effect sizes were small for most comparisons (Table S2).

Gingival thickness on the buccal side at the level of CEJ was significantly increased in all groups, regardless of the direction and magnitude of the tooth movement, but at a level of 1 mm apically from CEJ, no significant changes in gingival thickness were observed. On the lingual side, the gingiva thickness on both levels was significantly increased in the “Retro-inclination” group and reduced in the “Proclination-low” and “Proclination-high” groups. At both evaluated levels, a weak, significant negative correlation between the changes in gingiva thickness on the lingual side and individual inclination of lower incisors was observed (Figure 2B, r = −0.273 and r = −0.316). Most of the observed statistically significant differences in gingival thickness changes in the lower incisors during the first OT phase and between groups did not achieve a power level of 0.8, and the effect sizes were small to moderate (Table S2).

### 3.3. Relationship Between Changes in Soft Tissue Parameters and Vertical Bone Level During the First Phase of OT

**Figure 3:**
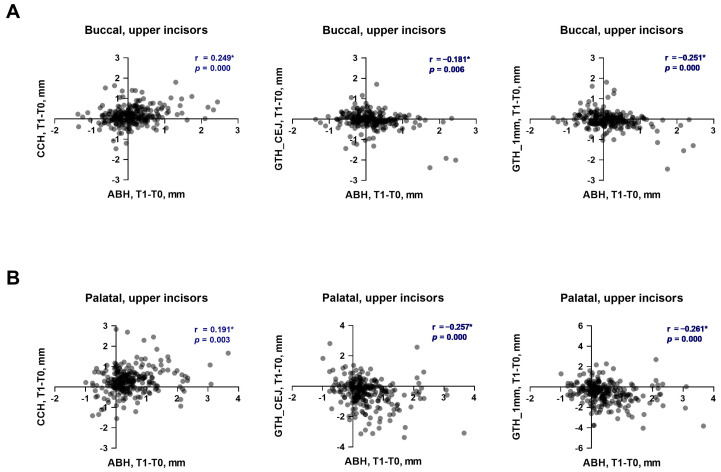
Relationship between the changes in soft tissue parameters and alveolar bone height during the first phase of orthodontic treatment in upper incisors. (**A**)—buccal side; (**B**)—palatal side. Each point represents the values measured on an individual tooth. For each pair of parameters, a correlation coefficient (r) and *p* values were calculated using the Spearman method. Correlations with *p* < 0.05 were considered to be statistically significant. *—statistically significant correlation.

**Figure 4:**
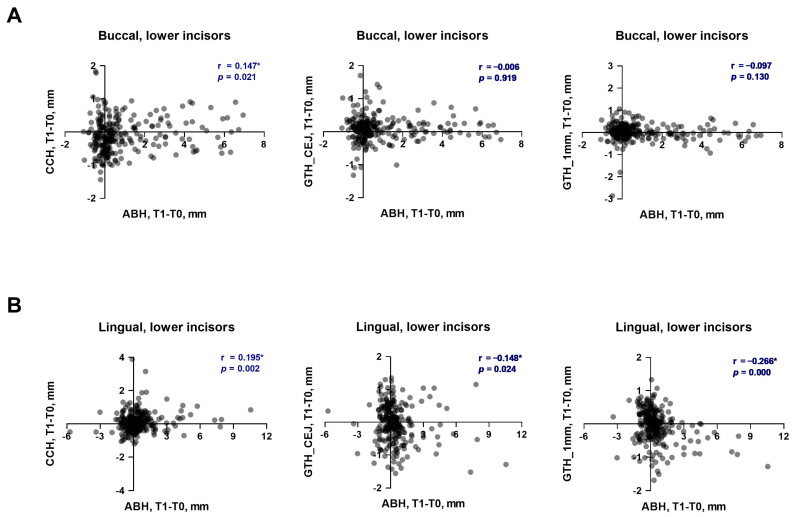
Correlation of changes in soft tissue parameters with alteration of vertical bone level during the first phase of orthodontic treatment in lower incisors. (**A**)—buccal side; (**B**)—lingual side. Each point represents the values measured on an individual tooth. For each pair of parameters, a correlation coefficient (r) and *p* values were calculated using the Spearman method. Correlations with *p* < 0.05 were considered to be statistically significant. *—statistically significant correlation.

The relationships between the changes in various soft tissue parameters and the vertical bone level during the first phase of OT in the upper and lower incisors are presented in Figure 3 and Figure 4, respectively. The changes in the vertical bone level of upper incisors on the buccal side had a weak significant positive correlation with CCH (r = 0.249) and a very weak to weak significant negative correlation with gingival thickness (r = −0.181 and r = −0.251) at both measured levels (Figure 3A). On the palatal side a very weak positive correlation of the vertical bone level with CCH (r = 0.191) and a weak correlation with gingiva thickness on both levels (r = −0.257 and r = −0.261) was found (Figure 3B). In lower incisors, a very weak significant positive correlation of the vertical bone level with CCH was found on both buccal (r = 0.147, Figure 4A) and lingual (r = 0.195, Figure 4B) sides. On the lingual side, a very weak to weak significant negative correlation between the vertical bone level and gingiva thickness was observed at both levels (r = −0.148 and r = −0.266, Figure 4B). On the buccal side of lower incisors, no correlation between the changes in the vertical bone level and gingiva thickness during the first phase of OT was observed (Figure 4A).

### 3.4. Relationship Between Changes in Clinical Crown Height and Gingival Thickness During the First Phase of OT

**Figure 5:**
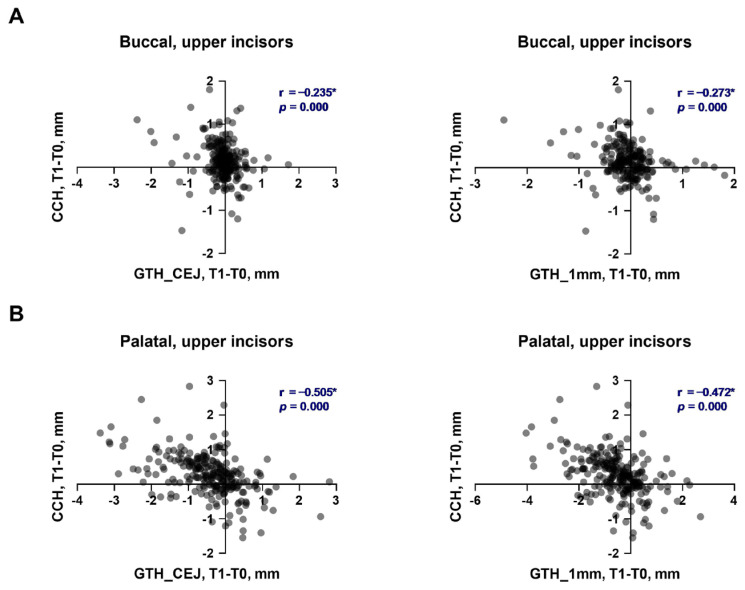
Correlation of changes in the clinical crown height and gingival thickness during the first phase of orthodontic treatment in upper incisors. (**A**)—buccal side; (**B**)—palatal side. Each point represents the values measured on an individual tooth. For each pair of parameters, a correlation coefficient (r) and *p* values were calculated using the Spearman method. Correlations with *p* < 0.05 were considered to be statistically significant. *—statistically significant correlation.

**Figure 6:**
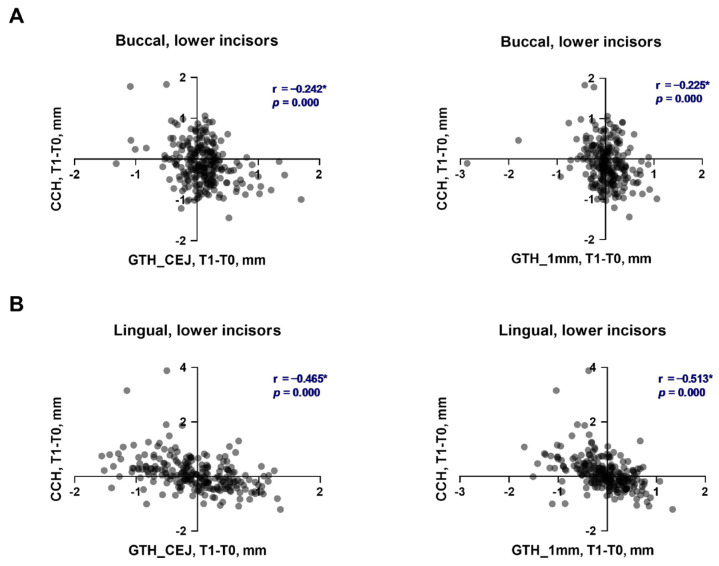
Correlation of changes in the clinical crown height and gingival thickness during the first phase of orthodontic treatment in lower incisors. (**A**)—buccal side; (**B**)—lingual side. Each point represents the values measured on an individual tooth. For each pair of parameters, a correlation coefficient (r) and *p* values were calculated using the Spearman method. Correlations with *p* < 0.05 were considered to be statistically significant. *—statistically significant correlation.

The relationships between the changes in clinical crown height and gingival thickness during the first phase of OT in the upper and lower incisors are presented in Figure 5 and Figure 6, respectively. The changes in CCH of upper incisors on both sides exhibited a negative correlation with the changes in gingiva thickness on both measured levels. The correlation was weak for the buccal side (r = −0.235 and r = −0.273, Figure 5A) and moderate for the palatal side (r = −0.505 and r = −0.472, Figure 5A). The changes in CCH of lower incisors on both sides exhibited a negative correlation with the changes in gingiva thickness on both measured levels. The correlation was weak for the buccal side (r = −0.242 and r = −0.273, Figure 5A) and moderate for the lingual side (r = −0.505 and r = −0.472, Figure 5A).

## 4. Discussion

Tooth displacement during OT occurs through the remodeling of the alveolar bone and gingiva and is sometimes accompanied by an apical migration of the marginal gingiva. Changes in incisor inclination during OT can alter the vertical and sagittal parameters of the gingiva [1,3,6,8,11,15]. In this study, we found that both gingival height and thickness were changed during the alignment phase of OT depending on the direction and magnitude of change in incisor inclination.

During the alignment phase of OT, there was a statistically significant increase in clinical crown height in the upper incisor region, indicating vertical gingival loss buccally and palatally. Similar results were obtained buccally in the upper incisors after OT when evaluating changes in CCH [12] and the position of the labial gingival margin relative to the CEJ [37]. The amount of inclination changes did not affect the increase in CCH buccally. In contrast, on the palatal side, changes in CCH depended on changes in tooth inclination: retro-inclination did not lead to significant gingival changes, but proclination resulted in a significant increase in CCH. Moreover, a statistically significant moderate correlation was observed between changes in inclination and CCH. Furthermore, the changes in CCH in the group with proclination higher than 5° were up to 0.6 mm, which is markedly higher than those observed on the buccal side (up to 0.2 mm). Thus, changes in tooth inclination during the first phase of OT result in gingiva loss, especially on the palatal side of the upper incisors. A clinically negligible (up to 0.2 mm) vertical loss of gingiva on the buccal side of the upper incisors did not depend on changes in inclination and could be due to the OT itself.

In lower incisors, changes in inclination resulted in both increases and decreases in CCH during the alignment phase of OT. However, the extent of these changes did not exceed 0.2 mm, and their clinical significance is questionable. Buccally, a decrease in CCH was observed independent of changes in inclination, indicating an increase in the gingival vertical level. These data contrast with another study, which measured GR using a caliper and noted apical gingival migration after the entire OT [1]. This discrepancy could be explained by the fact that changes were evaluated at different time points of OT. On the lingual side of the lower incisors, retro-inclination resulted in a significant decrease in CCH by less than 0.1 mm. In comparison, proclination led to a significant increase in CCH by up to 0.2 mm. Similar results regarding changes in vertical gingival level were observed in a study where a statistically significant increase in lingual CCH was found during OT [8]. We also observed a weak positive correlation between the changes in inclination and CCH on the lingual side. Thus, similarly to upper incisors, proclination of lower incisors during the first phase of OT results in vertical gingival loss, but this effect in lower incisors is not as pronounced as in the upper incisors.

Changes in the inclination of incisors during the alignment phase of OT impacted not only the change in gingival height but also thickness. Similarly to the changes in CCH, the most significant changes in the gingival thickness were observed on the palatal side of the upper incisors. Here, the teeth with a proclination of more than 5° exhibited a decrease in the gingival thickness of about 1 mm at the CEJ level and about 1.2 mm at the level of 1 mm below CEJ. Furthermore, a moderate, significant negative correlation was observed between changes in inclination and gingiva thickness on the palatal side of upper incisors. On the buccal side of the upper incisors, the first phase of OT resulted in a relatively small (less than 0.1 mm) decrease in gingiva thickness, which did not depend on the direction and magnitude of changes in inclination. The lack of dependence of the change in gingival thickness on inclination is consistent with previous studies [33,38].

In lower incisors, the changes in the gingiva thickness during the first phase of OT were rather small and did not exceed 0.2 mm at any side or level. On both the buccal and lingual sides, a very weak correlation between changes in the inclination and gingiva thickness was observed, suggesting that tooth proclination results in a decrease in gingiva thickness on both sides of the lower incisors. These data are consistent with the study by Zawawi and Al-Zahrani, which showed that a greater inclination of lower incisors after OT was associated with a higher prevalence of thin gingival biotype buccally [20]. The data from our study showed partial correlation with previous results, indicating a statistically significant decrease in gingival thickness after OT in the buccal areas of the upper and lower incisors. Other studies have not found a correlation between changes in lower incisor inclination and buccal gingival thickness [33,38].

It is worth noting that the changes in the vertical gingival level and thickness were interconnected, as indicated by the correlation analysis. Particularly, changes in CCH during the alignment phase of OT exhibited a negative correlation with the thickness at both measured levels. Thus, the vertical loss in the gingiva is always accompanied by a decrease in gingiva thickness. This observation confirms that there is a complex gingiva remodeling even during the first phase of OT.

The changes in the ABH are typical for the OT and are observed even during the alignment phase. We have observed a weak to very weak correlation between changes in the ABH and gingival parameters. Thus, the vertical bone loss on both sides of the upper and lower incisors correlated with the vertical gingiva loss and decrease in the gingiva thickness. Thus, the remodeling of bone and soft tissue during OT is interconnected.

During OT, dental plaque accumulation on the teeth increases, especially buccally, due to the presence of braces, which can lead to gingivitis [39]. Gingival inflammation leads to an increase in their volume and, in some cases, to hypertrophy. Mild hypertrophic gingivitis appeared buccally 1–2 months after bracket system fixation and persisted throughout the OT, even with good oral hygiene [40,41]. It is possible that gingivitis was responsible for the decrease in the buccal CCH in the lower incisor region and the lack of significant changes in the buccal CCH in the upper incisor region compared to the palatal/lingual changes. It could also have affected the buccal gingival thickness, especially at the GTH_CEJ level.

It is known that GR appears only in places of vertical defects of the alveolar bone. A weak positive correlation was obtained between the CCH and ABH buccally and palatally/lingually in the region of the upper and lower incisors. This implies a risk of gingival height loss associated with vertical alveolar bone loss. A weak negative correlation between ABH and GTH_CEJ and GTH_1mm is noted buccally and palatally in the region of the upper incisors and lingually in the region of the lower incisors.

It should be noted that, despite statistical significance, most changes in the gingival thickness and CCH found during the alignment phase of OT did not exceed 0.2 mm and could be considered as clinically non-significant. However, these changes cannot be neglected. It should be noted that we assessed alterations in gingival parameters only after the OT alignment phase, and the progression of these changes to the end of therapy cannot be excluded. We did not focus on GR itself, but an increase in CCH could be considered an initial phase of the GR; therefore, this parameter should be carefully controlled. The changes in the gingiva thickness higher than 0.2 mm were observed only on the palatal side after proclination with a higher amplitude. We did not specifically address how changes in gingival parameters depend on the gingival phenotype. However, these changes might be especially crucial in patients with a thin gingiva phenotype. Based on the data from this study and our previous publication, we recommend performing CBCT before orthodontic treatment in patients with a thin gingival and bone biotype to enable careful planning of tooth movement and to evaluate potential risks.

Limitations of this study include the lack of data on changes in gingival parameters at the end of OT and at follow-up. It is not known what happens with the gingiva during the second phase of OT and after, whether gingival remodeling occurs, increasing height and thickness over time, or increasing the volume of destruction. This study did not account for the vertical displacement of the incisors, which often accompanies a change in inclination. Changes in gingival parameters such as keratinized gingiva, attached gingiva, probing depth, or gingival inflammation were also not taken into account. Most of the correlations were weak to moderate and should be interpreted with caution. Finally, this study is limited to OT without premolar extraction and orthognathic surgery.

## 5. Conclusions

In summary, we found that the soft tissue changes during the alignment phase of OT depended on changes in inclination. This dependency is especially pronounced on the palatal/lingual side of both upper and lower incisors. Furthermore, the reduction in gingival height and thickness during the alignment phase was greater in the upper incisors. This difference may be due to the fact that the gingival thickness is greater in the palatal region of the upper incisors in comparison to the thickness in the lingual region of the lower incisors. It is also possible that this is due to the greater vertical bone loss during palatal OT [14]. Potential changes in the soft tissue should be considered when planning orthodontic treatment and when controlling them during the second phase of OT to avoid changes in the vertical gingival level and worsening gingival esthetics.

## Figures and Tables

**Table 1:** Change in the soft tissue parameters depending on the amount and direction of inclination alteration in upper incisors.

			Retro-Inclination	Proclination-Low	Proclination-High
Buccal	CCH, mm	T0	9.01 ± 1.05 (71)	8.59 ± 1.26 (90)	8.53 ± 1.16 (85)
		T1	9.14 ± 1.11 (71)	8.73 ± 1.22 (90)	8.72 ± 1.14 (85)
		T1 − T0	0.13 ± 0.48 (71) ^a^	0.14 ± 0.36 (90) ^a^	0.20 ± 0.49 (85) ^a^
	GTH_CEJ, mm	T0	1.54 ± 0.45 (64)	1.60 ± 0.36 (87)	1.66 ± 0.66 (81)
		T1	1.50 ± 0.33 (62)	1.52 ± 0.42 (87)	1.61 ± 0.40 (81)
		T1 − T0	−0.03 ± 0.38 (62)	−0.08 ± 0.37 (87)	−0.04 ± 0.53 (81)
	GTH_1mm, mm	T0	1.70 ± 0.41 (56)	1.76 ± 0.35 (78)	1.86 ± 0.60 (79)
		T1	1.66 ± 0.31 (56)	1.67 ± 0.36 (80)	1.79 ± 0.47 (80)
		T1 − T0	−0.03 ± 0.36 (56)	−0.05 ± 0.40 (80) ^a^	−0.05 ± 0.50 (80)
Palatal	CCH, mm	T0	8.74 ± 0.87 (71)	8.57 ± 1.04 (90)	8.22 ± 0.97 (85)
		T1	8.76 ± 0.84 (71)	8.79 ± 0.96 (90)	8.81 ± 1.01 (85)
		T1 − T0	0.02 ± 0.64 (71)	0.22 ± 0.52 (90) ^a,b^	0.59 ± 0.59 (85) ^a,c,d^
	GTH_CEJ, mm	T0	2.44 ± 0.85 (70)	2.44 ± 0.68 (90)	2.77 ± 0.90 (82)
		T1	2.42 ± 0.83 (70)	2.14 ± 0.70 (90)	1.85 ± 0.56 (82)
		T1 − T0	−0.02 ± 0.79 (70)	−0.30 ± 0.74 (90) ^a,b^	−0.92 ± 0.97 (82) ^a,c,d^
	GTH_1mm, mm	T0	3.22 ± 0.98 (70)	3.22 ± 0.75 (90)	3.60 ± 1.05 (79)
		T1	3.14 ± 0.98 (70)	2.81 ± 0.86 (90)	2.43 ± 0.80 (79)
		T1 − T0	−0.09 ± 0.79 (70)	−0.42 ± 0.78 (90) ^a,b^	−1.16 ± 1.20 (79) ^a,c,d^

Data are presented as mean ± SD (n); Abbreviations: T0, pre-orthodontic treatment; T1, after the alignment phase of OT. ^a^ Indicates significantly changed during the therapy (*p* < 0.05, Wilcoxon test). ^b^ Significantly different between “Proclination low” and the “Retro-inclination” groups (*p* < 0.05, Mann–Whitney U test). ^c^ Significantly different between “Proclination-high” and the “Retro-inclination” groups (*p* < 0.05, Mann–Whitney U test). ^d^ Significantly different between “Proclination-high” and the “Proclination low” groups (*p* < 0.05, Mann–Whitney U test).

**Table 2:** Change in the soft tissue parameters depending on the amount and direction of inclination alteration in lower incisors.

			Retro-Inclination	Proclination-Low	Proclination-High
Buccal	CCH, mm	T0	7.91 ± 0.80 (78)	8.01 ± 0.79 (86)	7.86 ± 0.91 (84)
		T1	7.84 ± 0.86 (78)	7.90 ± 0.84 (86)	7.73 ± 0.99 (84)
		T1 − T0	−0.07 ± 0.43 (78)	−0.11 ± 0.52 (86) ^a^	−0.14 ± 0.57 (84) ^a^
	GTH_CEJ, mm	T0	0.98 ± 0.29 (78)	1.08 ± 0.30 (85)	1.06 ± 0.42 (84)
		T1	1.09 ± 0.34 (78)	1.14 ± 0.32 (85)	1.18 ± 0.35 (84)
		T1 − T0	0.12 ± 0.30 (78) ^a^	0.05 ± 0.34 (85) ^a^	0.12± 0.46 (84) ^a^
	GTH_1mm, mm	T0	1.14 ± 0.26 (78)	1.20 ± 0.34 (84)	1.20 ± 0.36 (81)
		T1	1.14 ± 0.30 (78)	1.21 ± 0.26 (84)	1.25 ± 0.30 (81)
		T1 − T0	−0.00 ± 0.31 (78)	0.00 ± 0.42 (84)	0.06 ± 0.38 (81)
Palatal/ Lingual	CCH, mm	T0	8.55 ± 0.71 (78)	8.54 ± 0.70 (86)	8.29 ± 0.87 (84)
		T1	8.48 ± 0.78 (78)	8.70 ± 0.58 (86)	8.49 ± 0.71 (84)
		T1 − T0	−0.07 ± 0.43 (78) ^a^	0.16 ± 0.66 (86) ^a,b^	0.20 ± 0.66 (84) ^a,c^
	GTH_CEJ, mm	T0	0.86 ± 0.43 (73)	1.00 ± 0.47 (79)	0.93 ± 0.52 (80)
		T1	0.98 ± 0.48 (73)	0.93 ± 0.44 (79)	0.76 ± 0.53 (80)
		T1 − T0	0.12 ± 0.52 (73) ^a^	−0.07 ± 0.53 (79) ^b^	−0.17 ± 0.69 (80) ^a,c^
	GTH_1mm, mm	T0	1.14 ± 0.40 (74)	1.36 ± 0.45 (83)	1.34 ± 0.43 (82)
		T1	1.25 ± 0.31 (74)	1.27 ± 0.44 (83)	1.15 ± 0.47 (82)
		T1 − T0	0.11 ± 0.34 (74) ^a^	−0.09 ± 0.44 (83) ^a,b^	−0.19 ± 0.61 (82) ^a,c^

Data are presented as mean ± SD (n); Abbreviations: T0, pre-orthodontic treatment; T1, after the alignment phase of OT. ^a^ Indicates significantly changed during the therapy (*p* < 0.05, Wilcoxon test). ^b^ Significantly different between “Proclination low” and the “Retro-inclination” groups (*p* < 0.05, Mann–Whitney U test). ^c^ Significantly different between “Proclination-high” and the “Retro-inclination” groups (*p* < 0.05, Mann–Whitney U test).

## Data Availability

The original contributions presented in this study are included in the article. Further inquiries can be directed to the corresponding author.

## Supplementary Materials

The following supporting information can be downloaded at: https://www.mdpi.com/article/10.3390/dj13120587/s1, Figure S1: Method of automatic alignment of a DICOM file from CBCT and an STL file from an intraoral scan; Figure S2: Measuring the gingival parameters from merged CBCT and intraoral scan images; Table S1: Effect size and statistical power for various comparisons of the soft tissue parameter of upper incisors, which are presented in Table 1; Table S2: Effect size and statistical power for various comparisons of the soft tissue parameter of lower incisors, which are presented in Table 2.

## Abbreviations

The following abbreviations are used in this manuscript:

OT	Orthodontic treatment
ABH	Alveolar Bone Height
CCH	Clinical Crown Height
CEJ	Cement Enamel Junction
GTH	Gingival Thickness
GR	Gingival Recession
