# Supplementary material for: Relationship Between the Changes in the Inclination of the Incisors and Soft Gingival Tissue Remodeling During the First Phase of Orthodontic Treatment Without Premolar Extraction
Source: Dent J (Basel). 2025 Dec 8;13(12):587. doi: 10.3390/dj13120587 (PMC12731660; doi:10.3390/dj13120587)
Supplement: Supplementary file 1 [file dentistry-13-00587-s001.zip › dentistry-3937593-supplementary.pdf]

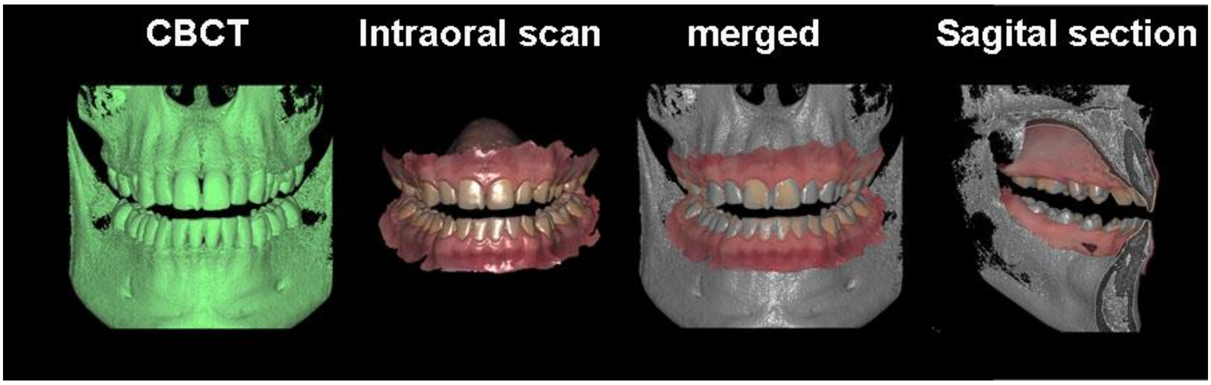

Supporting Figure S1. Method of automatic alignment of a DICOM file from CBCT and an STL file from an intraoral scan.

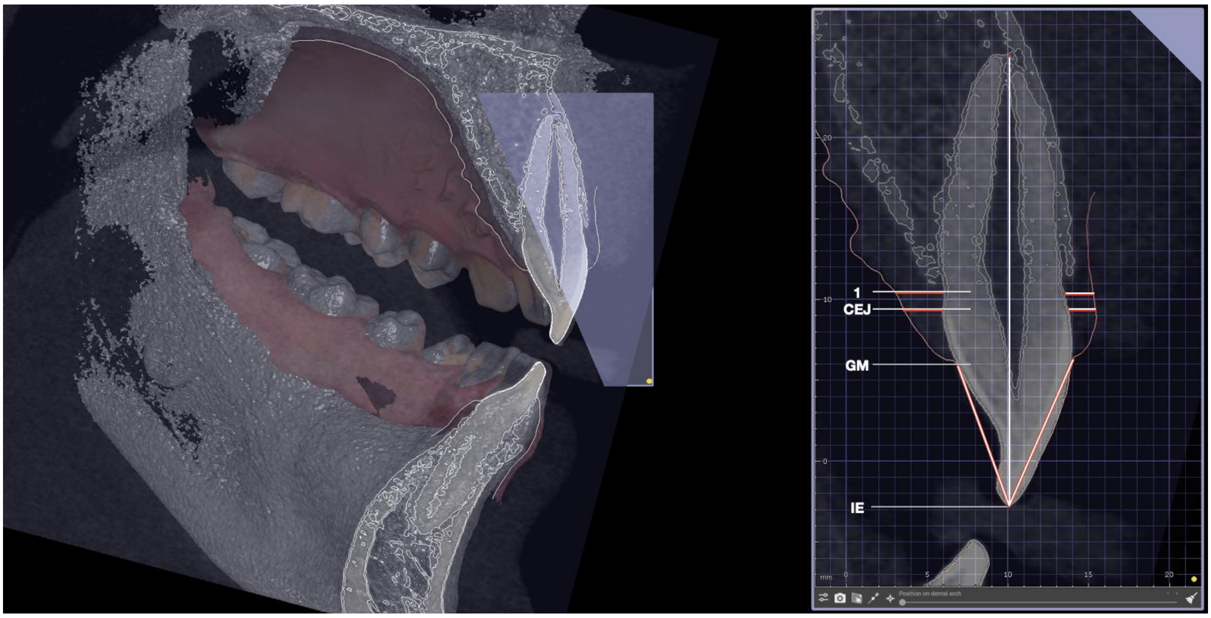

Supporting Figure S2. Measuring the gingival parameters from merged CBCT and intraoral scan images

Measurement clinical crown height (CCH) as a distance from incisal edge (IE) to point of the free gingival margin (GM) buccally and palatally/lingually and gingival thickness (GTH) perpendicularly to the long axis of upper and lower incisors at the level of cementoenamel junction (CEJ) and 1 mm apically from CEJ on both lingual and buccal sides of the tooth.

Supporting table S1. Effect size and statistical power for various comparisons of the soft tissue parameter of upper incisors, which are presented in Table 1.

Cohen's d was calculated to characterize the effect size. Statistical power (1 – β) was calculated at the level of α = 0.05. Parameters were calculated only for statistically significant differences. Statistically non-significant differences are marked as "n.s.".

		Buccal		Palatal	
		Cohen's d	(1 – β)	Cohen's d	(1 – β)
CCH	T1-T0, „Retro-inclination“	0.27;	0.61	n.s.	n.s.
	T1-T0, „Proclination-low“	0.39;	0.95	0.42	0.98
	T1-T0, "Proclination-high"	0.41;	0.96	1.00	1.00
	"Proclination-low" *vs.* "Retro-inclination"	n.s.	n.s.	0.34	0.55
	"Proclination-high" *vs.* "Retro-inclination"	n.s.	n.s.	0.93	1.00
	"Proclination-low" *vs.* "Proclination-high"	n.s.	n.s.	0.67	0.99
GTH_CEJ	T1-T0, „Retro-inclination“	n.s.	n.s.	n.s.	n.s.
	T1-T0, „Proclination-low“	n.s.	n.s.	0.41	0.97
	T1-T0, "Proclination-high“	n.s.	n.s.	0.95	1.00
	"Proclination-low" *vs.* "Retro-inclination"	n.s.	n.s.	0.37	0.60
	"Proclination-high *vs.* "Retroinclination"	n.s.	n.s.	1.02	1.00
	"Proclination low" *vs.* "Proclination-high"	n.s.	n.s.	0.72	0.99
GTH_1mm	T1-T0, „Retro-inclination“	n.s.	n.s.	n.s.	n.s.
	T1-T0, „Proclination-low“	0.13	0.20	0.54	0.99
	T1-T0, "Proclination-high"	n.s.	n.s.	0.97	1.00
	"Proclination-low“ *vs.* "Retro-inclination“	n.s.	n.s.	0.43	0.72
	"Proclination-high“ *vs.* "Retro-inclination“	n.s.	n.s.	1.05	1.00
	"Proclination-low“ *vs.* "Proclination-high“	n.s.	n.s.	0.73	0.99

Supporting table S2. Effect size and statistical power for various comparisons of the soft tissue parameter of lower incisors, which are presented in Table 2.

Cohen's d was calculated to characterize the effect size. Statistical power (1 – β) was calculated at the level of α = 0.05. Parameters were calculated only for statistically significant differences. Statistically non-significant differences are marked as "n.s.".

		Buccal		Lingual	
		Cohen's d	(1 – β)	Cohen's d	(1 – β)
CCH	T1-T0, „Retro-inclination"	n.s.	n.s.	0.16	0.29
	T1-T0, „Proclination-low"	0.21	0.49	0.24	0.60
	T1-T0, "Proclination-high"	0.25	0.60	0.30	0.78
	"Proclination-low" *vs.* "Retro-inclination"	n.s.	n.s.	0.41	0.72
	"Proclination-high" *vs.* "Retro-inclination"	n.s.	n.s.	0.48	0.84
	"Proclination-low" *vs.* "Proclination-high"	n.s.	n.s.	n.s.	n.s.
GTH_CEJ	T1-T0, „Retro-inclination"	0.40	0.94	0.23	0.49
	T1-T0, „Proclination-low"	0.15	0.27	n.s.	n.s.
	T1-T0, "Proclination-high"	0.26	0.66	0.24	0.59
	"Proclination-low" *vs.* "Retro-inclination"	n.s.	n.s.	0.36	0.58
	"Proclination-high *vs.* "Retroinclination"	n.s.	n.s.	0.47	0.80
	"Proclination low" *vs.* "Proclination-high"	n.s.	n.s.	n.s.	n.s.
GTH_1mm	T1-T0, „Retro-inclination"	n.s.	n.s.	0.32	0.78
	T1-T0, „Proclination-low"	n.s.	n.s.	0.20	0.45
	T1-T0, "Proclination-high"	n.s.	n.s.	0.31	0.80
	"Proclination-low" *vs.* "Retro-inclination"	n.s.	n.s.	0.51	0.86
	"Proclination-high" *vs.* "Retro-inclination"	n.s.	n.s.	0.61	0.95
	"Proclination-low" *vs.* "Proclination-high"	n.s.	n.s.	n.s.	n.s.
